# Increased Levels of Soluble ST2 in Patients with Active Newly Diagnosed ANCA-Associated Vasculitis

**DOI:** 10.1155/2015/603750

**Published:** 2015-02-23

**Authors:** Z. Hladinova, Z. Hruskova, B. Svobodova, K. Malickova, V. Lanska, P. Konopásek, E. Jancova, R. Rysava, C. L. Edelstein, V. Tesar

**Affiliations:** ^1^Department of Nephrology, General University Hospital and First Faculty of Medicine, Charles University, 121 08 Prague, Czech Republic; ^2^Institute of Immunology and Microbiology, General University Hospital and First Faculty of Medicine, Charles University, 121 08 Prague, Czech Republic; ^3^Institute of Medical Biochemistry and Laboratory Diagnostics, General University Hospital and First Faculty of Medicine, Charles University, 121 08 Prague, Czech Republic; ^4^Statistical Unit, Institute for Clinical and Experimental Medicine, 140 21 Prague, Czech Republic; ^5^Division of Renal Diseases and Hypertension, University of Colorado Denver, Aurora, CO 800 45, USA

## Abstract

*Objective*. ST2, a member of the interleukin-1 receptor family, is selectively expressed on Th2 cells and mediates important Th2 functions. IL-33 is a specific ligand of ST2. The aim of the study was to determine whether serum levels of soluble ST2 (sST2) or IL-33 predict activity of the disease in patients with ANCA-associated vasculitides (AAV). *Methods*. 139 AAV patients and 62 controls were studied. IL-33 and sST2 in the blood were measured with a commercially available ELISA. *Results*. Newly diagnosed AAV patients had higher sST2 levels than controls (*P* < 0.01). Levels of sST2 were significantly higher in active newly diagnosed AAV patients than in patients with remission (*P* < 0.001). IL-33 levels were higher in AAV patients than in the control groups (*P* = 0.002). However, serum IL-33 levels were not increased in patients with active AAV compared to patients in remission. IL-33 levels were higher in patients with granulomatosis with polyangiitis than in patients with microscopic polyangiitis (*P* = 0.012). *Conclusions*. Serum sST2, but not serum IL-33, may be a marker of activity in AAV patients.

## 1. Introduction

ANCA-associated vasculitides (AAV) are autoimmune diseases characterized by inflammation of the blood vessel wall and include microscopic polyangiitis (MPA), granulomatosis with polyangiitis (GPA, formerly Wegener's), and eosinophilic granulomatosis with polyangiitis (EGPA, formerly Churg-Strauss syndrome). Antineutrophil cytoplasmic antibodies (ANCA) can usually be detected in the blood of AAV patients. Cytoplasmic ANCA (cANCA), mainly directed against proteinase 3 (PR3-ANCA), predominate in GPA, and perinuclear ANCA (pANCA), directed against myeloperoxidase (MPO-ANCA), in MPA [1].

During the past few decades, significant advances have been made in understanding the disease pathogenesis and clinical management of AAV, with substantial decrease in mortality. During long-term follow-up, about half of the patients suffer from relapses [2]. Standard induction treatment still consists of cyclophosphamide or methotrexate and high doses of corticosteroids. The clinical status does not always correlate with the serum levels of ANCA. As there is a need to individualize the treatment, the emphasis has been placed on discovering new potential biomarkers of disease activity, which may help us to tailor the treatment better.

Interleukin-1 (IL-1) cytokines play an important role in immune regulation and inflammatory processes. IL-33 is a recently identified cytokine that is a member of the IL-1 family that also consists of IL-1 and IL-18 [3]. IL-33 is a dual function cytokine with extracellular and intracellular functions. The extracellular form of IL-33 activates a target cell by binding to the T1/ST2 receptor and induces Th2 associated cytokines but IL-33 also functions at intracellular level as a nuclear factor regulating transcription [3–5]. Recently, IL-33 has been ranked among “alarmins,” endogenous danger signals activating the immune system, released by necrotic but not apoptotic cells [5, 6]. IL-33 is expressed in endothelial cells and is a marker of endothelial cell quiescence [7]. IL-33 induces both proinflammatory and protective effects depending upon organ involved. IL-33 binds to ST2. ST2, a member of the interleukin-1 receptor family, is selectively expressed on Th2 (T helper type 2) cells and mediates important Th2 functions. There are two different forms of ST2: transmembrane form (ST2L) and secreted soluble form (sST2) [4]. Soluble ST2 is a negative regulator of IL-33 signalling pathway. Soluble ST2 is a decoy receptor for functional IL-33.

The IL-33/ST2 pathway has been the subject of a number of recent studies [8–15]. Elevated sST2 levels have been found in patients with several cardiovascular and/or pulmonary diseases (asthma, acute dyspnoea, and myocardial infarction), but also sepsis, and elevated sST2 levels are considered an unfavourable prognostic marker in these patients [9]. IL-33 levels were increased in patients with disease associated with chronic inflammation such as ankylosing spondylitis [10], rheumatoid arthritis [11], Henoch-Schonlein purpura [12], or multiple sclerosis [13]. In SLE patients elevated sST2 levels were recently described to correlate with disease activity [9]. Low levels of IL-33 and high levels of sST2 were noted in patients with HIV infection [14] or amyotrophic lateral sclerosis [15].

The aim of this study was to determine whether serum levels of IL-33 or ST2 are markers of disease activity in patients with AAV, a disease characterised by endothelial injury and chronic inflammation.

## 2. Patients and Controls

The study included 165 samples from 139 AAV patients (67 men, 72 women) recruited from the Department of Nephrology, General University Hospital, Prague, Czech Republic, during a 15-month period. All patients gave informed consent. For patient characteristics see Tables 1 and 2.

Diagnoses in this study were assessed using the EMA algorithm [16]. Patients with EGPA were excluded. The disease activity was assessed with BVAS (Birmingham Vasculitis Activity Score version 3 [17]), with the use of clinical data and laboratory tests routinely performed (erythrocyturia, proteinuria, and serum levels of ANCA, CRP, and S-creatinine).

Active disease was defined as BVAS ≥ 1 and remission as BVAS = 0. Relapse was defined as a new/worse disease activity and BVAS ≥ 1 after previous remission. Persistent activity was defined as no new/worse activity, with persistent BVAS ≥ 1. In total, 38 samples were from patients with active disease (16 active newly diagnosed, 11 active with relapse, and 11 persistent activity) and 127 samples were from patients in remission that were available for examination. Twenty-one patients were sampled repeatedly but within each subgroup all patients were unique. Samples from newly diagnosed patients were collected immediately after the diagnosis of AAV had been confirmed but some of the patients had already started immunosuppressive treatment.

The control group contained 62 patients (34 men, 28 women): 22 healthy individuals and 40 patients with chronic kidney disease (median of creatinine 168 *μ*mol/L in nondialysis patients; 19x hemodialysis patients, 8x IgA nephropathy, 10x polycystic kidney disease, and 3x other).

After obtaining blood samples, all patients were routinely followed up in the clinics and clinical activity (relapses) and deaths were recorded for the purpose of this study.

### 2.1. Laboratory Methods

A total of 10 mL of blood were collected from all subjects. Serum samples were stored at −80°C until analysed. Serum IL-33 and sST2 levels were measured with a commercially available ELISA (enzyme-linked immunosorbent assay) kit. The sST2 ELISA kit was obtained from MBL International, Woburn, MA, USA (Code number 7638). The human serum IL-33 ELISA kit was obtained from R&D Systems Inc., Minneapolis, MN, USA (Product number DY3625).

### 2.2. Statistical Analysis

Software SYSTAT 10 was used. The chi square test was used for comparison of discrete variables; if the minimum expected value was less than 5, the Yates correction was applied. For continuous variables, the Kruskal-Wallis test with multiple comparison or the Mann-Whitney test in the case of two groups was used. The association between variables was measured by Spearman's rank correlation. All tests were two sided and *P* < 0.05 was considered statistically significant.

## 3. Results

Ranges and medians of ST2 and IL-33 levels are shown in Table 3.

**Figure 1:**
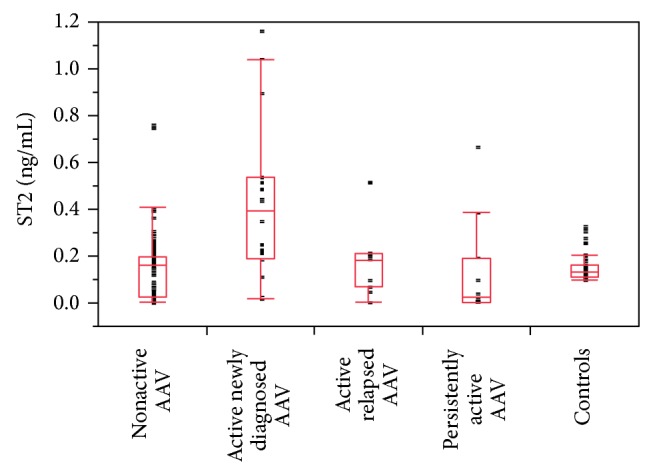
Comparison of sST2 levels in AAV patients with different disease activity stages and controls. ST2 levels were higher in active newly diagnosed AAV versus nonactive AAV (*P* < 0.001), persistently active AAV: *P* < 0.001, and controls (*P* < 0.01). No other significant differences were found. Box plot graph: boxes represent 25–75 percentile (50% of results in the box = interquartile range (IQR), bottom is the first quartile, top is the third quartile, and median is represented by the line). Whiskers are lines extending vertically from the boxes, which indicate variability outside the upper and lower quartiles. Results which are not included between the whiskers are plotted as an outlier with a dot.

Levels of sST2 in AAV patients did not differ from renal and healthy controls. The subgroup of patients with active newly diagnosed AAV had higher median sST2 levels than healthy and renal controls (median 0.39 ng/mL versus 0.13 ng/mL and *P* < 0.01, Table 3, Figure 1). Median levels of sST2 in patients with active AAV tended to be higher compared to patients in remission but the difference did not reach statistical significance. Subsequent analysis revealed that AAV patients with active newly diagnosed disease have higher sST2 levels than AAV patients in remission (*P* < 0.001, Figure 1). On the contrary sST2 levels in patients with relapse or persistent activity did not significantly differ from remission.

Nine of 12 patients, who were sampled repeatedly in different phases of disease (active newly diagnosed AAV → remission, or remission → relapse), had lower levels of sST2 in remission than in active disease stage (Table 4).

Levels of IL-33 were significantly higher in all AAV patients, in active AAV, and in AAV in remission than in healthy and renal control patients (*P* = 0.002, *P* = 0.007, and *P* = 0.007, resp.). Levels of IL-33 tended to be lower in patients with active AAV than in patients in remission but the difference did not reach statistical significance (median IL-33 levels = 0 pg/mL in both groups, mean IL-33 levels are 13.16 pg/mL in active AAV patients versus 41.79 pg/mL in patients with remission, *P* = 0.119).

No difference in sST2 or IL-33 levels was found between female and male patients. No relation to levels of ANCA, BVAS, S-creatinine, CRP, and proteinuria was disclosed. In the group of newly diagnosed patients there was no difference in group with serum creatinine above 300 *μ*mol/L (8 patients) and below 300 *μ*mol/L (8 patients) (median ST2 levels 0.463 ng/mL and 0.221 ng/mL, resp., *P* = 0.667). In the group of newly diagnosed patients there was no difference in ST2 levels between patients who already initiated immunosuppressive treatment (9 patients) and those without treatment (7 patients) at the time of blood sampling (median ST2 levels 0.434 ng/mL and 0.350 ng/mL, resp., *P* = 0.425). In the group of newly diagnosed patients there was no difference in ST2 levels in patients with higher BVAS (BVAS ≥ 15, 7 patients) and lower BVAS (BVAS < 15, 9 patients) (median ST2 levels 0.214 ng/mL and 0.343 ng/mL, resp., *P* = 0.382). All newly diagnosed patients had IL-33 levels 0 ng/mL. There was no difference between PR3-ANCA and MPO-ANCA but levels of IL-33 in patients who suffered from granulomatosis with polyangiitis were higher than IL-33 levels in patients with microscopic polyangiitis (*P* = 0.012). Levels of IL-33 were higher in younger AAV patients than older patients (*P* = 0.007). No significant difference in sST2 and IL-33 levels was found between patients with and without lung involvement (median sST2 levels in 0.17 ng/mL and 0.13 ng/mL, resp., *P* = 0.338, median IL-33 levels = 0 pg/mL in both groups).

The median length of follow-up (FU) after blood collection was 30 (range 2–50) months. During the FU, 22 patients (15.8%) died and 60 patients (43.2%), 38 PR3-ANCA and 22 MPO-ANCA, suffered from a relapse. We did not observe any difference in ST2 or IL-33 levels between patients who relapsed and those without relapse (median 0.14 ng/mL versus 0.17 ng/mL and *P* = 0.181) and between patients who died and the surviving patients (median sST2 0.16 ng/mL versus 0.16 ng/mL, *P* = 0.650; median IL-33 levels = 0 pg/mL in both groups).

## 4. Discussion

Elevated levels of sST2 are present in the circulation of patients with various inflammatory diseases. The proinflammatory cytokine IL-33 is the ligand for ST2 [18]. To our knowledge, this is the first study to examine the association between serum levels of IL-33 and sST2 in AAV patients and disease activity. Similar to a previous study in SLE patients [9], we demonstrated higher sST2 levels in active newly diagnosed AAV patients compared to healthy controls. Elevated sST2 levels in patients with active vasculitis may suggest a relationship of sST2 with the inflammatory process in AAV. However, while the study in SLE showed a correlation of sST2 levels with disease activity, we were not able to demonstrate association of sST2 with any other markers of disease activity, including ANCA levels, CRP, or BVAS.

Contrary to the levels of serum ST2, there was no difference in IL-33 in patients with active AAV compared to patients in remission. In theory, this might be caused by the lower production and/or secretion of IL-33 or by its rapid clearance. There are a growing number of publications describing the precise function of this intriguing novel cytokine that has attracted a lot of attention lately but the cellular source of IL-33 is less clear [5]. Nevertheless, it seems that IL-33 is mainly expressed in quiescent endothelial cells and the expression is lost when contact inhibition is disrupted or cells are exposed to proinflammatory IL1-*β* or TNF-*α* [5, 7] which are processes known to participate in neutrophil priming in early pathogenesis phases of AAV. Subsequently, when remission is achieved and endothelial damage repaired, IL-33 levels might increase, which was also noted in this study even though the results did not reach statistical significance. Whether endothelial cells are the dominant IL-33 secreting cells in vivo is, however, uncertain. Interestingly, the situation in an immune-complex vasculitis, Henoch-Schonlein purpura (HSP), was just the opposite; the authors described elevated IL-33 levels in acute HSP that decreased in remission [12]. We were not able to demonstrate any statistically significant difference in different disease activity subgroups of AAV patients in this study, but this might be caused by low sensitivity of the assays used.

Active IL-33 is known to be degraded by the proapoptotic caspase-3 [19]. Another explanation for lack of increase in IL-33 levels in active AAV might be that IL-33 is subject to degradation by caspases released from the apoptotic cells in AAV, in accordance with the hypothesis that was suggested for amyotrophic lateral sclerosis [15]. Neutrophil priming and apoptosis have been described in AAV [20]. Primed neutrophils undergo apoptosis more readily, at which times they express PR3 and MPO on their surface. It is possible that degradation of IL-33 by proapoptotic caspase-3 released from primed neutrophils in active AAV may explain the decreased levels of IL-33. As the serum receptor for IL-33, sST2 molecule, was increased in active AAV patients, it is conceivable that sST2 acts as a contraregulating agent, regulating inflammatory process [15].

There are possible limitations of the study. Most patients in this study have renal involvement. Levels of ST2 and IL-33 may behave differently in patients with extrarenal involvement, which would require further studies. A few of the newly diagnosed patients had received immunosuppressive treatment prior to blood collection, which may, in general, influence levels of circulating biomarkers in AAV [21]. However, we were not able to find any difference in IL-33 levels and ST2 levels between the already treated and untreated newly diagnosed patients at the time of blood sampling. The serum levels of sST2 and particularly IL-33 were low and we were not able to detect measurable IL-33 levels in any of the control patients. Thus, a more sensitive assay might be needed for routine detection. Alternately, intracellular IL-33 expression and production may not result in a robust increase in serum IL-33 levels and IL-33 as with some other cytokines may have a very short half-life. Analysis of tissue IL-33 expression, for example, in kidney biopsies might provide important clues to reveal the role of IL-33/ST2 pathway in the pathogenesis of AAV, which was, however, not an objective in this study. It might be useful to confirm the results in a longitudinal prospective study but the limited number of repeated measurements performed in patients in this study is in accordance with the cross-sectional results.

## 5. Conclusion

In conclusion, results of this pilot study suggest that sST2 may be a marker of activity in active newly diagnosed patients with AAV. Further studies with serial samples and a prospective follow-up are needed to elucidate the precise role of sST2/IL-33 in AAV.

## Figures and Tables

**Table 1:** Basic clinical characteristics of patients with AAV.

Characteristics of patients with AAV		
Number of patients	139	
Age	Median 59 years	Range 20–84 years
Gender	M/F 67/72	48.2%/51.8%
Diagnosis		
Granulomatosis with polyangiitis (GPA)	73	52,5%
Microscopic polyangiitis (MPA)	66	47.5%
ANCA-ELISA result (ever)		
Anti-PR3	75	54,0%
Anti-MPO	61	43,9%
Negative	3	2.1%
Disease duration	Median 35 months	Range 0–231 months
Lung involvement (ever)	86 patients	61.9%
Kidney involvement (ever)	138 patients	99.2%

**Table 2:** Laboratory characteristics.

Total number of samples = 165		
Erythrocyturia (at the time of blood sampling)		
Positive (number of samples)	44	26.7%
Negative (number of samples)	110	67.0%
Not available	11	6.3%
Proteinuria	Median 0.27 g/day	Range 0.04–13.68 g/day
CRP	Median 3.9 mg/L	Range 1–314.7 mg/L
Serum creatinine	Median 154 *μ*mol/L	Range 53–640 *μ*mol/L
	18x dialysis	

**Table 3:** Ranges and medians of sST2 levels and IL-33 levels in AAV patients.

Group of patients (number of samples)	sST2 ng/mL		IL-33 (ng/mL)	
	Median	Range	Median	Range
All AAV (165)	0.16	0–1.17	0	0–2.60
Active AAV (38)	0.19	0–1.17	0	0–0.29
Active newly diagnosed AAV (16)	0.39	0–1.17	0	0.00
Remission AAV (127)	0.17	0–0.76	0	0–2.60
Control group (62)	0.13	0.10–10.15	0	0.00

**Table 4:** ST2 levels in patients, who were sampled repeatedly in different phases of disease (active newly diagnosed AAV → remission, or remission → relapse) (*higher levels displayed in bold*).

ST2 levels (ng/mL) in active AAV	ST2 levels (ng/mL) in remission
**0.18**	0.16
**0.44**	0.21
**0.54**	0.26
**0.90**	0.40
**0.19**	0.17
**0.10**	0.04
**0.48**	0.20
**0.15**	0.09
**0.03**	0.01
**0.07**	0.06
0.18	**0.19**
0.18	**0.19**
0.05	**0.19**

## References

[1] Jennette J. C., Falk R. J. (1997). Small-vessel vasculitis. *The New England Journal of Medicine*.

[2] Walsh M., Flossmann O., Berden A. (2012). Risk factors for relapse of antineutrophil cytoplasmic antibody-associated vasculitis. *Arthritis and Rheumatism*.

[3] Arend W. P., Palmer G., Gabay C. (2008). IL-1, IL-18, and IL-33 families of cytokines. *Immunological Reviews*.

[4] Kakkar R., Lee R. T. (2008). The IL-33/ST2 pathway: therapeutic target and novel biomarker. *Nature Reviews Drug Discovery*.

[5] Haraldsen G., Balogh J., Pollheimer J., Sponheim J., Küchler A. M. (2009). Interleukin-33—cytokine of dual function or novel alarmin?. *Trends in Immunology*.

[6] Zhao W., Hu Z. (2010). The enigmatic processing and secretion of interleukin-33. *Cellular and Molecular Immunology*.

[7] Sundlisæter E., Edelmann R. J., Hol J. (2012). The alarmin IL-33 is a notch target in quiescent endothelial cells. *The American Journal of Pathology*.

[8] Arshad M. I., Piquet-Pellorce C., Samson M. (2012). IL-33 and HMGB1 alarmins: sensors of cellular death and their involvement in liver pathology. *Liver International*.

[9] Mok M. Y., Huang F. P., Ip W. K. (2009). Serum levels of IL-33 and soluble ST2 and their association with disease activity in systemic lupus erythematosus. *Rheumatology*.

[10] Han G. W., Zen L. W., Liang C. X. (2011). Serum levels of IL-33 is increased in patients with ankylosing spondylitis. *Clinical Rheumatology*.

[11] Hong Y.-S., Moon S.-J., Joo Y.-B. (2011). Measurement of interleukin-33 (IL-33) and IL-33 receptors (sST2 and ST2L) in patients with rheumatoid arthritis. *Journal of Korean Medical Science*.

[12] Chen T., Jia R. Z., Guo Z. P., Cao N., Li M. M., Jiao X. Y. (2013). Elevated serum interleukin-33 levels in patients with Henoch-Schönlein purpura. *Archives of Dermatological Research*.

[13] Christophi G. P., Gruber R. C., Panos M., Christophi R. L., Jubelt B., Massa P. T. (2012). Interleukin-33 upregulation in peripheral leukocytes and CNS of multiple sclerosis patients. *Clinical Immunology*.

[14] Miyagaki T., Sugaya M., Yokobayashi H. (2011). High levels of soluble ST2 and low levels of IL-33 in sera of patients with HIV infection. *Journal of Investigative Dermatology*.

[15] Lin C. Y., Pfluger C. M., Henderson R. D., McCombe P. A. (2012). Reduced levels of interleukin 33 and increased levels of soluble ST2 in subjects with amyotrophic lateral sclerosis. *Journal of Neuroimmunology*.

[16] Watts R., Lane S., Hanslik T. (2007). Development and validation of a consensus methodology for the classification of the ANCA-associated vasculitides and polyarteritis nodosa for epidemiological studies. *Annals of the Rheumatic Diseases*.

[17] Suppiah R., Mukhtyar C., Flossmann O. (2011). A cross-sectional study of the Birmingham vasculitis activity score version 3 in systemic vasculitis. *Rheumatology*.

[18] Dinarello C. A. (2005). An IL-1 family member requires caspase-1 processing and signals through the ST2 receptor. *Immunity*.

[19] Lüthi A. U., Cullen S. P., McNeela E. A. (2009). Supression of interleukin-33 bioactivity through proteolysis by apoptotic caspases. *Immunity*.

[20] Harper L., Cockwell P., Adu D., Savage C. O. S. (2001). Neutrophil priming and apoptosis in anti-neutrophil cytoplasmic autoantibody-associated vasculitis. *Kidney International*.

[21] Monach P. A., Kümpers P., Lukasz A. (2012). Circulating angiopoietin-2 as a biomarker in ANCA-associated vasculitis. *PLoS ONE*.

